# Prescription drug monitoring programs and prescription pain medication misuse among U.S. high school students—2019

**DOI:** 10.1186/s12889-024-18698-1

**Published:** 2024-05-10

**Authors:** Kevin Liu, Marco Benedetti, Alexander Evans, Motao Zhu

**Affiliations:** 1 Center for Injury Research and Policy, Abigail Wexner Research Institute at Nationwide Children’s Hospital, Columbus, OH USA; 2 Division of Epidemiology, College of Public Health, The Ohio State University, Columbus, OH USA; 3 Department of Pediatrics, College of Medicine, The Ohio State University, Columbus, OH USA

**Keywords:** Prescription drug monitoring programs, Prescription pain medication misuse, Youth risk behavior survey

## Abstract

**Background:**

Prescription drug monitoring programs (PDMPs) are state-level databases that track and inform prescribing practices to reduce prescription drug diversion and misuse. To our knowledge, only three studies have examined the impact of PDMPs on opioid-related outcomes among adolescents, and none have focused on prescription pain medication misuse among adolescents.

**Methods:**

This study leveraged data from the 2019 National Youth Risk Behavior Survey (YRBS) to explore the associations between five categories of PDMP dimensions and the prevalence of self-reported prescription pain medication misuse. Demographic factors’ associations with self-reported prescription pain medication misuse were also examined.

**Results:**

In 2019, none of the PDMP dimensions were associated with self-reported prescription pain medication misuse among U.S. high school students, adjusting for gender, grade, race/ethnicity, and sexual orientation.

**Conclusions:**

None of the five PDMP dimensions were associated with lower prescription pain medication misuse, however further research is needed, especially as new YRBS data become available.

## Background

More than 14% of US high school students reported ever misusing a prescription pain medication in 2019, increasing their risk for heroin initiation, injection drug use, and development of opioid use disorder later in life [1]. Adolescent prescription pain medication misuse has also been linked to other risk behaviors including driving behaviors like texting while driving, seat belt use, and driving while intoxicated; violent behaviors like carrying a weapon and involvement in physical fights; sexual behaviors like increased number of sexual partners and unprotected sex; and use of other substances like alcohol and tobacco [2]. Prescription pain medication misuse in adolescence has also been linked to heroin initiation and injection drug use in later adolescence and early adulthood [3, 4]. Despite widespread implementation of prescription drug monitoring programs (PDMPs) in response to the ongoing opioid epidemic, their impacts on opioid-related outcomes are not yet well understood, particularly among adolescents [5].PDMPs are state-level systems designed to reduce prescription drug misuse by tracking prescribing practices to inform clinical practice. Finley et al. [6] proposed a conceptual framework for PDMPs, where data reporting leads to increased monitoring that affects opioid prescribing behaviors, thereby acting on opioid-related outcomes like volume of prescribed opioids, opioid misuse, and opioid-related overdoses and deaths. As state-level interventions, PDMP dimensions like the drug schedules that are tracked, users who have access, and reasons or requirements for use vary by state. A limited evidence base suggests that proactive PDMP dimensions, like sending users unsolicited reports of potential misuse, may be most important for a PDMP to be effective [7].

The majority of studies on PDMP effectiveness have focused on other opioid-related outcomes among adults, such as opioid prescribing behaviors or opioid-related mordbidity or mortality. A previous study evaluating the effects of PDMPs on nonmedical use of prescription opioids in adults showed no effect on the prevalence of prescription opioid misuse, but did show an association with a reduction in the number of days of misuse in the past year [8].

The lack of studies focused onadolescents who misuse prescription opioids typically obtain their drugs from friends or relatives rather than directly from prescribers and dispensers in the health care system [9]. This could suggest PDMPs may be less effective at reducing prescription pain medication misuse among adolescents when compared with older age groups. However, it may be possible for these policies to have indirect effects on prescription pain medication misuse among adolescents by limiting the supply of drugs prescribed to their friends or family members.

To our knowledge, only three studies have examined the impact of PDMPs on opioid-related outcomes among adolescents, and none have focused on prescription pain medication misuse among adolescents. Earlywine, Hadland & Raifman reported that PDMPs with mandated prescriber use significantly reduced injection drug use among 17–18 year olds [10]; Toce et al. found significant long-term decreases in opioid poisoning rates among those aged 0–4 years, 5–9 years, and 15–19 years following PDMP implementation [11]; and Theodorou et al. reported no statistically significant change in the rate of postoperative opioid prescriptions for California patients aged less than 18 years after a change to the state’s PDMP in 2018 [12].

Research on how PDMPs associate with teen opioid use and misuse is relatively recent, as are some of the self-reported data that allow researchers to better understand the association. In 2019, the Youth Risk Behavior Survey asked a question about prescription pain medication misuse. In the present study, we examined the associations between five categories of PDMP dimensions and the prevalence of self-reported prescription pain medication misuse among U.S. high school students. We hypothesize that unsolicited reports to prescribers, unsolicited reports to dispensers, and mandatory use for prescribers will be associated with lower prescription pain medication misuse.

## Methods

The data set consisted of 8,677 respondents to the 2019 National Youth Behavior Risk Survey (YRBS). Although the full data consisted of 13,872 respondents, some states chose to administer a survey that did not ask about our outcome variable. The response rate for the 2019 YRBS was 60.3% [13]. The outcome variable, called “prescription pain medication misuse” for shorthand, was derived from the following survey question, which was added to YRBS in 2019:

*During the past 30 days, how many times have you taken prescription pain medicine without a doctor’s prescription or differently than how a doctor told you to use it?*

We grouped responses into three categories: 1–2 times, 3–9 times, and 10 or more times. Also, like Jones et al., binary indicators of past-month prescription pain medication misuse were reported [14].

We cross-tabulated prescription pain medication misuse by gender, race/ethnicity, grade, sexual orientation, and PDMP dimensions. We identified five PDMP dimensions for analysis. These were based on eight dimensions identified by Cerda et al. [7], however we consolidated them into the following categories based on similarities in their intent and states’ concordance in enacting them:

1. Unsolicited reports to law enforcement (local, state, and federal vs. not).
2. Unsolicited reports to prescribers (yes vs. no).
3. Mandatory PDMP use for prescribers (yes vs. no).
4. Unsolicited reports to dispensers – individuals or businesses (yes vs. no).
5. Unsolicited reports to licensing bodies (yes vs. no).

PDMP dimension data were obtained from the Prescription Drug Monitoring Program Training and Technical Assistance Center (PDMP TTAC) at the Institute for Intergovernmental Research [15]. The PDMP TTAC regularly surveys PDMP administrators to collect data, publish state profile, and facilitate the sharing of best practices. For each state, each of the five dimensions were classified as present or absent in 2019.

Policy dimensions’ associations with self-reported prescription pain medication misuse were measured by fitting modified Poisson regression models in which the outcome was a binary indicator of the outcome, with a value of 1 denoting at least once instance of misuse. We choose Poisson regression over logistic regression because the estimated prevalence ratios provide for a more intuitive interpretation than odds ratios in this setting, and because odds ratios suffer from non-collapsibility [16]. Each model adjusted for gender, grade, race/ethnicity, and sexual orientation. Separate models were fit for each of the five dimensions listed above. The analyses were conducted using survey weighted procedures with SAS (version 9.4; SAS Institute).

## Results

Among the 25 states in our study sample, 23 (92%) had mandatory use for prescribers, 21 (84%) had PDMPs with unsolicited reports to prescribers, 15 (60%) had unsolicited reports to dispensers, 13 (52%) had unsolicited reports to licensing bodies, and 7 (28%) had unsolicited reports to local, state, and federal law enforcement agencies.

Table 1 presents cross-tabulations of self-reported prescription pain medication misuse by sex, grade, race/ethnicity, and sexual orientation. Female students reported higher prescription pain medication misuse than males (8.3% vs. 6.1%), and the sex difference is most pronounced for reporting misusing prescription pain medications 1–2 times in the last month (5.0% vs. 2.7%). Hispanic students and non-heterosexual students self-reported prescription pain medication misuse 1–2 times in the last month more than their counterparts. While there was little difference in the more frequent misuse categories (1–2 times and 3–9 times), gay, lesbian, and bisexual students and those who were unsure about their sexuality self-reported at least one instance of prescription pain medication misuse more often than heterosexual students. There was no apparent pattern to self-reported prescription pain medication misuse by grade level.

**Table 1:** Cross-tabulations of self-reported prevalence of prescription pain medication misuse by demographics

Variable	Level	Number asked about use of non-prescribed pain med.	Weighted percent	Percent who self-reported using non-prescribed pain medication in last 30 days			
				1–2 times	3–9 times	10 + times	At least once
Overall	NA	8677	100.0	3.9 (3.3, 4.4)	1.7 (1.2, 2.1)	1.7 (1.2, 2.2)	7.2 (6.3, 8.2)
Sex	Female	4379	49.6	5.0 (4.3, 5.8)	1.8 (1.3, 2.4)	1.5 (0.9, 2.1)	8.3 (7.0, 9.7)
	Male	4230	50.4	2.7 (2.2, 3.2)	1.5 (1.0, 2.1)	1.9 (1.2, 2.5)	6.1 (5.2, 7.0)
Grade	9th	2256	26.9	4.2 (3.1, 5.3)	1.4 (0.6, 2.2)	1.5 (0.7, 2.3)	7.1 (5.3, 8.8)
	10th	2338	25.5	3.6 (2.6, 4.5)	1.4 (0.7, 2.0)	2.0 (1.3, 2.8)	7.0 (5.4, 8.5)
	11th	2096	23.9	4.0 (3.2, 4.8)	2.2 (1.3, 3.1)	1.6 (1.0, 2.3)	7.8 (6.4, 9.2)
	12th	1918	23.6	3.7 (2.5, 4.8)	1.7 (1.0, 2.5)	1.4 (0.7, 2.1)	6.8 (5.1, 8.6)
Race/ ethnicity	NH* White	4169	52.5	3.0 (2.3, 3.7)	1.2 (0.9, 1.6)	1.3 (0.5, 2.1)	5.5 (4.3, 6.8)
	NH Black	1089	9.8	3.7 (2.7, 4.8)	2.3 (1.2, 3.5)	2.6 (1.6, 3.7)	8.7 (6.6, 10.8)
	Hispanic	2269	27.7	5.8 (4.9, 6.7)	1.9 (0.7, 3.0)	2.1 (1.4, 2.9)	9.8 (8.2, 11.4)
	NH Other	1043	10.0	3.1 (1.9, 4.3)	2.6 (1.2, 4.0)	1.4 (0.6, 2.3)	7.1 (5.6, 8.7)
Sexual orientation	Heterosexual	6863	84.8	3.6 (2.9, 4.3)	1.5 (1.1, 1.9)	1.2 (0.8, 1.7)	6.4 (5.4, 7.4)
	Gay, lesbian, bisexual	930	11.0	5.2 (3.9, 6.5)	3.4 (1.8, 5.0)	3.4 (1.6, 5.3)	12.0 (9.4, 14.6)
	Not sure	345	4.1	5.7 (3.1, 8.3)	1.1 (0.2, 2.0)	4.7 (1.7, 7.7)	11.5 (7.7, 15.3)

* NH = Non-Hispanic.

Table 2 presents cross-tabulations of self-reported prescription pain medication misuse by PDMP dimensions. There was little policy-based difference in self-reported prescription pain medication misuse. Table 2 also presents results from modified Poisson regression models. No PDMP dimensions were statistically significantly associated with self-reported prescription pain medication misuse.

**Table 2:** Cross-tabulation of self-reported prevalence of prescription pain medication misuse and adjusted prevalence ratios (PR) for PDMP policy dimensions

		Self-reported use at least once in last 30 days	Adjusted PR* (95% CI)	*p*-value
Unsolicited reporting to federal, state, and local law enforcement	Absent	7.2 (6.0, 8.5)		
	Present	7.2 (5.6, 8.9)	0.95 (0.70, 1.29)	0.740
Unsolicited reports to licensing bodies	Absent	7.3 (5.8, 8.8)		
	Present	7.0 (5.8, 8.1)	0.96 (0.73, 1.26)	0.756
Unsolicited reports to prescriber	Absent	6.7 (5.6, 7.7)		
	Present	7.3 (6.1, 8.4)	0.96 (0.70, 1.31)	0.792
Prescriber must access	Absent	6.1 (5.2,7.0)		
	Present	7.3 (6.2, 8.3)	1.06 (0.74, 1.51)	0.741
Unsolicited reports to dispensers	Absent	6.4 (5.3, 7.6)		
	Present	7.9 (6.6, 9.2)	1.13 (0.90, 1.41)	0.293

* All estimates adjusted for gender, grade, race/ethnicity, and sexual orientation

## Discussion

We conducted descriptive and regression analyses to examine associations between demographic factors with self-reported prescription pain medication misuse, and between five categories of PDMP policy dimensions with self-reported prescription pain medication misuse, among U.S. high school students. Some demographic patterns of self-reported prescription pain medication misuse were consistent with findings from previous studies, whereas others were not. For instance, female students reported a higher prevalence of prescription pain medication misuse than male students. This is consistent with results from a previous study on nonmedical prescription pain medication misuse among U.S. adolescents [17].

The current study’s finding that prescription pain medication misuse was least prevalent among heterosexual students is also consistent with previous studies [18]. However, the current study found that self-reported prescription pain medication misuse was least prevalent among non-Hispanic white students compared with non-Hispanic Black and Hispanic students. This is opposite of the findings from a previous survey on lifetime nonmedical opioid use among US high school seniors by Palamar et al. [19].

When cross-tabulations of self-reported prescription pain medication misuse by PDMP dimension were conducted, no evidence of associations between any PDMP dimensions and self-reported prescription pain medication misuse were discovered. We came to the same conclusion after adjusting for potential confounders. However, that is not to say these dimensions hold no effect in curbing the teen opioid epidemic. Most PDMPs have evolved since their initiation, adding different dimensions on different timelines from other PDMPs. As a result, some dimensions may have yet to influence prescription pain medication misuse. In addition, many teens who misuse prescription opioids obtain their drugs from friends or relatives [9]. The PDMPs may have trouble tracking these secondary exchanges. Several factors likely underlie PDMP efficacy, therefore continued research to better understand their effects on youth opioid misuse is greatly recommended.

Direct comparison of our PDMP-related findings with those from previous studies is made difficult by differences in study design, study populations, and outcome definitions. Ali et al. [6] examined the effects of PDMPs on prescription opioid misuse in adults and found no effect on past-year misuse, dependence, or initiation; though they did detect a reduction in the number of days of past-year misuse. Previous studies focused on adolescents have shown potential benefits with respect to other opioid-related outcomes, such as injection drug use [10] and opioid poisoning rates [11].

This study has several limitations. First, YRBS data are self-reported and respondents may provide socially desirable responses to questions about prescription pain medication misuse. Second, having only one year of data meant that our study cannot be used to draw conclusions about PDMP efficacy. It is plausible that existing differences in states, which were not controlled for in our model, underlie the observed relationships between PDMP dimensions and prescription pain medication misuse. This point is underscored by the fact that only four states in our study did not have unsolicited reporting to prescribers and only two did not require mandatory use for prescribers. When more waves of YRBS data become available, analyzing multiple years of data will yield more robust evidence for or against PDMP effects on teen opioid misuse. Third, this study did not account for the length of time PDMP dimensions have been in place, only whether the dimension was present or absent in 2019.

## Data Availability

Data from the 2019 national Youth Risk Behavior Survey are openly available online from the Centers for Disease Control and Prevention: https://www.cdc.gov/healthyyouth/data/yrbs/data.htm.

## Abbreviations

PDMP: Prescription drug monitoring program
YRBS: Youth Risk Behavior Survey
